# Detection and Clinical Significance of Chromosomal Mosaicism in Prenatal Diagnosis: A Retrospective Study From a Prenatal Diagnosis Center

**DOI:** 10.1002/jcla.70305

**Published:** 2026-08-10

**Authors:** Yuanyuan Pei, Xiaojin Luo, Jian Ran, Liang Hu, Weiqiang Liu, Fengxiang Wei

**Affiliations:** ^1^ The Genetics Laboratory, Maternity & Child Healthcare Hospital of Longgang District Shenzhen City (Longgang Maternity and Child Institute of Shantou University Medical College) Shenzhen Guangdong China; ^2^ Longgang District Maternity and Child Health and Intelligent Precision Medicine Shenzhen Guangdong China

**Keywords:** chromosomal mosaicism, genetic counseling, pregnancy outcomes, prenatal diagnosis, prenatal screening

## Abstract

**Background:**

To evaluate the detection capabilities of prenatal screening and diagnostic methods for fetal chromosomal mosaicism and analyze associated pregnancy outcomes.

**Methods:**

This retrospective study included 79 cases of fetal chromosomal mosaicism identified by karyotyping among 8106 women undergoing prenatal diagnosis. Sensitivity of screening methods (first‐trimester serum screening, second‐trimester serum screening, and noninvasive prenatal testing (NIPT)) and concordance of diagnostic methods (chromosomal microarray analysis (CMA), multiple STR locus analysis technique (QF–PCR)) with karyotyping were assessed. Pregnancy outcomes were analyzed.

**Results:**

NIPT demonstrated significantly higher sensitivity (90.74%) compared with first‐trimester serum screening (57.41%) and second‐trimester serum screening (39.29%) (*p* < 0.001). CMA showed high concordance with karyotyping (90.00%), whereas QF‐PCR exhibited significantly lower concordance (58.21%) (*p* < 0.001). The pregnancy termination rate was 57.14% (40/70). All trisomy 21 and trisomy 13 mosaicism cases resulted in termination, while decisions regarding other autosomal and sex chromosome mosaicism were more variable.

**Conclusion:**

NIPT outperforms traditional serum screening for detecting chromosomal mosaicism, though karyotyping remains essential for confirmation. CMA aligns well with karyotyping, particularly for low‐level mosaicism. The high termination rate underscores the critical need for multidisciplinary genetic counseling integrating genetic, imaging, and clinical data.

## Graphical abstract

This retrospective study of 79 prenatal mosaicism cases found that noninvasive prenatal testing (NIPT) had significantly higher sensitivity (90.74%) than first‐ and second‐trimester serum screening, while chromosomal microarray analysis (CMA) showed better concordance with karyotyping (90.00%) than QF‐PCR (58.21%). The high pregnancy termination rate (57.14%) underscores the critical need for multidisciplinary genetic counseling integrating genetic, imaging, and clinical data.

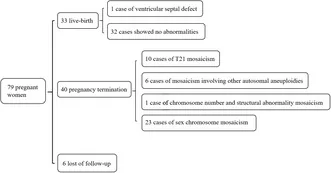

## 1 Introduction

Chromosomal disorders are a leading cause of congenital disabilities and intellectual impairments in newborns. Chromosomal mosaicism, a subtype arising from errors during cell division, results in the coexistence of two or more cell lines with distinct chromosomal compositions within an individual. Its clinical manifestations vary widely, depending on the affected tissues, chromosomes involved, and the proportion of abnormal cells, making each case unique. This complexity poses significant challenges for prenatal diagnosis and genetic counseling [1, 2].

Current prenatal screening methods for chromosomal abnormalities include serum screening, noninvasive prenatal testing (NIPT), ultrasound examination, and other methods. Diagnostic techniques comprise karyotyping, chromosomal microarray analysis (CMA), multiple STR locus analysis technique (QF–PCR), fluorescence in situ hybridization (FISH), and other methods [3, 4]. Serum screening and NIPT are mainly used for common aneuploidy screening; their efficacy in mosaicism detection is unclear. At present, studies on the combined application of karyotyping and molecular techniques in mosaicism diagnosis are insufficient [5].

This study aims to evaluate the detection capabilities of various prenatal screening and diagnostic methods for chromosomal mosaicism by comparing their performance against the gold standard of karyotyping. By analyzing associated pregnancy outcomes, we seek to provide evidence to inform clinical decision‐making and genetic counseling.

## 2 Materials and Methods

### 2.1 Study Design and Population

This retrospective study included pregnant women who underwent prenatal diagnosis at the Prenatal Diagnosis Center of Maternity & Child Healthcare Hospital of Longgang District Shenzhen City between January 2019 and December 2023. Inclusion criteria were a diagnosis of fetal chromosomal mosaicism via karyotype analysis. Cases with pure chromosomal aneuploidy or structural abnormalities were excluded. Mosaicism was confirmed by karyotyping, with reporting of levels as low as 3% based on standard laboratory practice, acknowledging that levels below 10% may have uncertain clinical relevance. All participants provided informed consent, and the study was approved by the hospital ethics committee.

Among 8106 women who underwent prenatal diagnosis during the study period, 79 with fetal chromosomal mosaicism were identified and included. The screening and diagnostic results of the 79 mosaic cases are detailed in the Results section. To avoid gender identification for non‐medical needs, all fetal sex chromosomes are denoted as XN.

### 2.2 Specimen Collection

Specimens were collected according to standard clinical protocols: blood samples for serum screening (at 9^+0^–13^+6^ and 15^+0^–20^+6^ weeks) and NIPT (after 12 weeks); chorionic villus sampling (CVS) at 11–13^+6^ weeks; amniocentesis at 16–26^+6^ weeks; and cordocentesis after 22 weeks. All CMA and QF‐PCR tests were performed on the same specimen as karyotyping.

### 2.3 Prenatal Screening and Diagnostic Methods

The methodologies for serum screening, NIPT, karyotyping, CMA, and QF‐PCR were performed as previously described [6], with key parameters summarized below.

#### 2.3.1 Serum Screening

First‐trimester screening combined free β‐hCG, PAPP‐A, and nuchal translucency (NT) to assess T21/T18 risk. Second‐trimester screening used AFP, free β‐hCG, and uE3 for T21/T18/neural tube defects (NTDs) risk. The high‐risk cutoff for T21 was 1:270, with moderate risk being 1:270–1:1000. For T18, the high‐risk cutoff was 1:350, with moderate risk being 1:350–1:500.

#### 2.3.2 NIPT

Performed on the BGISEQ500 platform, analyzing cfDNA from maternal plasma. A *Z*‐score > 3 indicated high risk for T21, T18, or T13.

#### 2.3.3 Karyotyping

Cultured cells from CVS, amniotic fluid, or cord blood were G‐banded (320–400 band resolution). A minimum of 20 karyotypes were analyzed, expanded to 100 if mosaicism was suspected. Although karyotype analysis can detect mosaicism without a specific lower limit, given the potential clinical significance of low‐level mosaicism, mosaicism was reported at levels ≥ 3%. Karyotypes were described according to ISCN 2016 guidelines.

#### 2.3.4 CMA

The Affymetrix Cytoscan 750K array was used, detecting unbalanced rearrangements and numerical abnormalities at a resolution of ≥ 100 Kb. The test detects mosaicism reliably at levels > 30%, with a lower limit of approximately 10%.

#### 2.3.5 QF–PCR

Multiplex STR genotyping for chromosomes 13, 18, 21, X, and Y was performed on the ABI 3500Dx Genetic Analyzer. The test reliably identifies mosaicism at a 20% level, with a detection limit as low as 10%.

### 2.4 Statistical Analysis

Statistical analysis was performed using R software (version 4.1.0). Continuous variables were presented as mean ± standard deviation, and categorical variables as numbers and percentages (%). The sensitivity of different screening methods (first‐trimester serum screening, second‐trimester serum screening, NIPT) was calculated using karyotyping as the reference. The concordance rates of diagnostic methods (CMA, QF‐PCR) with karyotyping were also calculated. For all proportions, 95% confidence intervals (CI) were computed. Differences in detection rates between screening methods, and in concordance rates between CMA and QF‐PCR, were analyzed using the *χ* ^2^ test. A *p* value of less than 0.05 was considered statistically significant. No corrections for multiple testing were applied, as the primary comparisons were pre‐specified.

## 3 Results

### 3.1 Participant Profiles

The overall incidence of mosaicism among women undergoing prenatal diagnosis was 0.97% (79/8106, 95% CI: 0.76%–1.19%). For amniotic fluid specimens, the detection rate of mosaicism was 0.92% (68/7363, 95% CI: 0.74%–1.19%). Maternal age, gestational age, sampling methods, chromosomal distribution, and mosaicism ratios were detailed in Table 1. Sex chromosome mosaicism was the most common type (51/79, 64.56%, 95% CI: 53.82%–75.30%). Three patients with chromosomal aneuploidy mosaicism in chorionic villi had normal karyotypes in subsequent amniotic fluid, indicating confined placental mosaicism (CPM).

**TABLE 1:** Characteristics of the study subjects.

Clinical characteristics	Data	Percentages of different chromosomal categories (%)
Samples (*n*)	79	/
Age (years)	30.7 ± 5.22	/
Gestational week (weeks)	17.97 ± 3.23	/
Specimen		
Chorionic villus	10	/
Amniotic fluid	71^a^	/
Umbilical cord blood	1	/
Autosomal mosaicism		
Chromosomal distribution in aneuploid mosaicism		
chr.21	11^b^	13.92
chr.9	5	6.33
chr.13	2	2.53
chr.7	2^b^	2.53
chr.22	1	1.27
chr.8	1	1.27
mar	1	1.27
Chromosomal structural abnormality mosaicism	3	3.80
Mosaicism with both chromosomal numerical and structural abnormalities	2	2.53
Sex chromosome mosaicism	51^b^	64.56
Mosaicism ratio		
< 10%	15	18.99
10%–29%	21	26.58
30%–49%	14	17.72
≥ 50%	29	36.71

^a^ Chorionic villus and amniotic fluid were both tested in three cases.

^b^ In one case each, chorionic villus showed mosaicism but amniotic fluid was normal (CPM). The percentages are calculated based on the total number of mosaic cases (*N* = 79).

### 3.2 Screening Results

Of the 54 cases that underwent first‐trimester serum screening, 31 (31/54, 57.41%, 95% CI: 43.75%–70.15%) were classified as high‐ or moderate‐risk, and 23 were low‐risk (6 with abnormal markers). Among the 28 who underwent second‐trimester serum screening, 11 (11/28, 39.29%, 95% CI: 23.59%–57.29%) were high‐ or moderate‐risk, and 17 were low‐risk. NIPT was performed in 54 cases, of which 49 (49/54, 90.74%, 95% CI: 80.69%–95.71%) showed high‐risk or other abnormalities. NIPT demonstrated significantly higher sensitivity compared to first‐trimester and second‐trimester serum screening (*χ* ^2^ = 37.21, *p* < 0.001). Detailed screening results are summarized in Table 2. The distributions of risk categories are illustrated in Figures [Link], [Link], [Link].

**TABLE 2:** Sensitivity of different screening methods for detecting chromosomal mosaicism (using karyotyping as reference).

Screening method	Number screened (*n*)	Positive screening results (*n*)	Sensitivity (%)	95% CI (%)
First‐trimester serum	54	31	57.41	43.75–70.15
Second‐trimester serum	28	11	39.29	23.59–57.29
NIPT	54	49	90.74	80.69–95.71

Twenty‐three pregnant women underwent all three screening methods. In two of these cases, all three screens were low‐risk, but mosaicism (47,XN,+9[10]/46,XN[90] and 45,X[45]/46,XN[22]) was subsequently diagnosed by karyotyping and confirmed by CMA.

### 3.3 Diagnostic Results and Concordance With Karyotyping

All 79 cases were diagnosed by karyotyping. Among the 70 cases that also underwent CMA, abnormalities were detected in 63, yielding a concordance rate of 90.00% (63/70, 95% CI: 81.21%–94.99%) with karyotyping (Table 3). The seven cases with discordant CMA results (all normal by CMA) had karyotypes showing mosaicism for trisomy 8 (mosaic ratio 8.0%), trisomy 7 (16.0%), structural abnormalities of chromosomes 20 (28.6%) and chromosome 7 (30.0%), translocations (17.8%, 42.9%), or a marker chromosome.

**TABLE 3:** Concordance of diagnostic methods with karyotyping for detecting chromosomal mosaicism.

Diagnostic method	Number of tested (*n*)	Concordant with karyotyping (*n*)	Concordance rate (%)	95% CI (%)
CMA	70	63	90.00	81.21–94.99
QF‐PCR	67	39	58.21	46.13–69.46
Karyotyping	79	79	/	/

Among the 67 cases that underwent QF‐PCR, abnormalities were detected in 39, resulting in a concordance rate of 58.21% (39/67, 95% CI: 46.13%–69.46%) with karyotyping (Table 3). The 28 discordant cases (normal by QF‐PCR) included two with T21 mosaicism (mosaic levels 9.8% and 56.7%) and 14 with sex chromosome mosaicism (mosaic levels ranging from 6.0% to 40.0%). *χ* ^2^ test revealed significant differences between CMA and QF‐PCR (*χ* ^2^ = 22.73, *p* < 0.001).

### 3.4 Pregnancy Outcomes

Pregnancy outcomes were available for 73 of the 79 cases (6 lost to follow‐up). Of these, 33 resulted in live births and 40 in termination of pregnancy. Excluding three cases of CPM, the termination rate among confirmed or likely true fetal mosaicism cases was 57.14% (40/70, 95% CI: 45.71%–67.93%) (Figure S4). All cases of trisomy 21 (*n* = 10) and trisomy 13 (*n* = 2) mosaicism resulted in termination. Among the 50 sex chromosome mosaicism cases, 46.00% (23/50, 95% CI: 32.19%–59.81%) were terminated. No clear correlation between mosaic proportion and the decision to terminate was observed.

Detailed case information, including karyotyping, mosaic ratio (percentage of abnormal cells by karyotyping), ultrasound findings, and pregnancy outcomes for all 79 cases, is provided in Table S1.

## 4 Discussion

The screening, diagnosis, prognosis, and recurrence risk assessment of mosaicism remain challenging in clinical practice, particularly in prenatal diagnosis [7]. Although there are some consensus guidelines for the classification and diagnosis of mosaicism, these standards primarily address diseases in children or adults and are not directly applicable to prenatal diagnosis or fetal medicine [8, 9]. Genetic counseling and clinical management of prenatal mosaicism are often challenged by either over‐intervention or under‐intervention, which not only increases the workload for laboratory and fetal medicine professionals but also causes anxiety and distress for pregnant women and their families. This retrospective study from a single prenatal diagnosis center provides a comprehensive evaluation of screening and diagnostic methods for fetal chromosomal mosaicism, comparing their performance against the gold standard of karyotyping. Our findings reaffirm the diagnostic challenges posed by mosaicism and offer practical insights for laboratory interpretation and genetic counseling.

Currently, the most common mosaicism in the general population is sex chromosome mosaicism, whereas other chromosomal mosaicism is rarely reported [10]. In this study, the incidence of mosaicism was 0.97%, with a significant number of sex chromosome mosaicism, which is consistent with the findings of previous studies [11]. The incidence of mosaicism in amniotic fluid karyotype analysis was 0.92%, which was higher than that reported in the literature [5, 12], likely due to our laboratory's practice of reporting low‐level mosaicism (≥ 3%). This underscores the importance of standardized reporting guidelines in prenatal settings, which currently lag behind those for pediatric or adult populations. Notably, in this study, CPM was strongly suspected in three cases where initial chorionic villus sampling (CVS) revealed chromosomal aneuploidy mosaicism (47,XN,+21[5]/46,XN[46]; 47,XN,+7[15]/46,XN[75]; 45,X[52]/46,XN[26]), but subsequent amniocentesis results were normal. CPM refers to the inconsistency between the chromosomal composition of the placenta and that of the fetus itself. There are cell lines with chromosomal numerical abnormalities (such as aneuploidy) in the placental cells, while the fetal cells themselves have a normal chromosomal composition (or the proportion of abnormalities is much lower than that in the placenta). These cases underscore the critical importance of follow‐up amniocentesis for accurate fetal diagnosis when mosaicism is detected by CVS.

In evaluating screening methods, NIPT demonstrated significantly higher sensitivity (90.74%) than first‐ or second‐trimester serum screening, confirming its superior role in prenatal screening for aneuploidies, including mosaicism [13]. This advantage stems from its direct analysis of placental cfDNA. However, NIPT is not designed to detect mosaicism definitively. The five false‐negative NIPT cases in our study highlight the key limitation: if the abnormal cell line is underrepresented in the placenta, the cfDNA fraction may fall below the detection threshold [13]. Therefore, a positive NIPT result can raise suspicion for mosaicism even without ultrasound structural abnormalities, indicating the need for prenatal diagnosis using multiple techniques; a negative NIPT result does not rule it out, and clinicians should continue routine prenatal care accordingly.

Regarding diagnostic confirmation, our results underscore the continued essential role of karyotyping. It remains the gold standard for visualizing and quantifying mosaic cell lines without a predefined lower limit. CMA showed high concordance (90.00%) with karyotyping, successfully detecting most cases except for some low‐level mosaicism and balanced structural rearrangements [14, 15]. In contrast, QF‐PCR demonstrated significantly lower concordance (58.21%), predominantly missing cases that exceeded its reliable detection threshold [16]. FISH can serve as a valuable adjunct, as it allows rapid detection of mosaicism without cell culture and may identify low‐level abnormal cell lines that could be missed by routine analysis. This reinforces the recommendation that a diagnosis of true mosaicism should ideally be based on concordant findings from at least two different techniques, and any diagnosis based on a single technique should be interpreted cautiously in the clinical context [17, 18].

The high rate of pregnancy termination (57.14%) in our cohort reflects the significant anxiety and uncertainty surrounding a prenatal diagnosis of mosaicism. All cases of trisomy 21 and 13 mosaicism were terminated regardless of ultrasound findings, whereas the termination decision for other autosomal and sex chromosome mosaicism cases was more complex. Among the cases with available records, neither other autosomal nor sex chromosome mosaicism cases exhibited obvious structural anomalies on prenatal ultrasound. This observation suggests that prenatal diagnosis is strongly recommended when ultrasound abnormalities are present, and the absence of ultrasound abnormalities does not rule out the possibility of mosaicism. The decision to proceed with invasive prenatal diagnosis and termination decisions should not be based solely on ultrasound findings. This highlights the critical need for comprehensive and multidisciplinary genetic counseling that integrates cytogenetic/molecular data, prenatal imaging, and up‐to‐date information on potential outcomes [19, 20].

This study has several limitations. First, its retrospective design introduces potential selection bias, as the cohort was derived from women already undergoing invasive testing. Second, the absence of high‐quality prenatal FISH data precluded a more integrated analysis of factors influencing pregnancy outcomes. Third, the inclusion of cases with low‐level mosaicism (< 10%), which is generally regarded as normal variation or culture artifact by some guidelines. Most importantly, the lack of postnatal follow‐up and phenotypic confirmation for live‐born infants is a significant limitation. We are therefore unable to correlate the level or type of prenatal mosaicism with actual clinical outcomes, which is the ultimate goal of prenatal diagnosis. Future prospective studies incorporating detailed prenatal phenotyping, postnatal follow‐up, and advanced technologies like single‐cell sequencing are needed to validate our findings and refine prognostic accuracy. Unlike conventional bulk analysis of cell populations, single‐cell approaches enable direct assessment of individual cells, allowing precise discrimination of true fetal mosaicism from CPM and cell culture illusion [21]. Cell‐based NIPT combined with single‐cell sequencing has demonstrated clinical feasibility for detecting aneuploidies and mosaicism by directly analyzing fetal cells enriched from maternal blood [22].

In conclusion, NIPT demonstrates superior sensitivity to traditional serum screening for detecting chromosomal mosaicism, though its limitations, particularly for low‐level mosaicism and CPM, must be recognized. Karyotyping remains essential for confirmation and characterization. CMA shows high concordance with karyotyping. The high rate of pregnancy termination underscores the profound uncertainty faced by families and the critical need for individualized, multidisciplinary counseling that integrates all available genetic and clinical data.

Based on our findings, we propose the following diagnostic workflow for suspected prenatal mosaicism: First, when karyotyping reveals a mosaic result, confirmation using an independent technique is recommended. Second, if mosaicism is detected on chorionic villus sampling, follow‐up amniocentesis should be strongly considered to rule out CPM. Third, NIPT‐positive results should be confirmed by diagnostic testing using confirmatory techniques such as karyotyping, CMA, QF‐PCR, or FISH; a negative NIPT does not exclude mosaicism. Fourth, prenatal genetic counseling should integrate cytogenetic results, molecular genetic results, and ultrasound findings to guide informed decision‐making. Finally, postnatal follow‐up is essential to correlate prenatal findings with postnatal phenotype and developmental outcomes.

## Author Contributions

All the authors materially participated in this study and manuscript preparation. Yuanyuan Pei collected all the clinical data and drafted the manuscript. Xiaojin Luo, Jian Ran, Liang Hu, and Weiqiang Liu carried out all the biochemical, molecular, and cytogenetic analyses. Fengxiang Wei designed the work and revised the manuscript. All authors read and approved the final manuscript.

## Funding

The study was supported by the Foundation of Longgang District Key Laboratory of Birth Defects Prevention (No. LGKCZSYS2018000010).

## Ethics Statement

Approval was granted by the Ethics Committee of Maternity & Child Healthcare Hospital of Longgang District, Shenzhen City (Longgang Maternity and Child Institute of Shantou University Medical College) (No: LGFYYXLLL‐2022‐024).

## Consent

The authors have nothing to report.

## Conflicts of Interest

The authors declare no conflicts of interest.

## Supporting information


**Figure S1:** First‐trimester serum screening results of the 54 pregnant women with fetal chromosomal mosaicism. ^†^4 cases were high‐risk for both T21 and T18, ^‡^1 case chorionic villus showed sex chromosome mosaicism but amniotic fluid was normal (CPM).

**Figure S2:** Second‐trimester serum screening results of the 28 pregnant women with fetal chromosomal mosaicism.

**Figure S3:** NIPT results of the 54 pregnant women with mosaic fetuses.

**Figure S4:** Pregnancy outcomes of the 79 pregnant women with fetal chromosomal mosaicism.

**Table S1:** Summary of Karyotyping, ultrasound findings, and pregnancy outcomes of the 79 pregnant women with fetal chromosomal mosaicism.

## Data Availability

The datasets used and/or analyzed during the current study are available from the corresponding author on reasonable request.
