# Supplementary material for: Detection and Clinical Significance of Chromosomal Mosaicism in Prenatal Diagnosis: A Retrospective Study From a Prenatal Diagnosis Center
Source: J Clin Lab Anal. 2026 Aug 10;40(16):e70305. doi: 10.1002/jcla.70305 (PMC13455827; doi:10.1002/jcla.70305)
Supplement: Supplementary file 1 [file JCLA-40-e70305-s001.docx]

| **Supplementary Table S1 Summary of Karyotyping, ultrasound findings, and pregnancy outcomes of the 79 pregnant women with fetal chromosomal mosaicism** | | | | | |
| --- | --- | --- | --- | --- | --- |
| Case ID | Gestational Weeks at Prenatal Diagnosis | Karyotyping | Mosaic Ratio (percentage of abnormal cells by karyotyping, %) | Ultrasound Findings | Pregnancy Outcome |
| 1 | 16 | 47,XN,+13[3]/46,XN[50] | 6.0 | no abnormalities detected | termination of pregnancy |
| 2 | 18 | 47,XN,+21[6]/46,XN[44] | 12.0 | no abnormalities detected | termination of pregnancy |
| 3^a^ | 13 | 47，XN,+21[5]/46,XN[46] | 9.8 | one of the twins stops developing | live birth |
|  | 16 | 46,XN | / |  |  |
| 4 | 17 | 47,XN,+21[34]/46,XN[26] | 56.7 | no abnormalities detected | termination of pregnancy |
| 5 | 20 | 47,XN,+21[44]/46,XN[18] | 70.9 | no abnormalities detected | termination of pregnancy |
| 6 | 12 | 47,XN,+21[5]/46,XN[52] | 8.8 | no abnormalities detected | termination of pregnancy |
| 7 | 16 | 47,XN,+21[18]/46,XN[32] | 36.0 | no abnormalities detected | termination of pregnancy |
| 8 | 15 | 47,XN,+21[50]/46,XN[6] | 89.3 | left choroid plexus cyst | termination of pregnancy |
| 9 | 16 | 47,XN,+21[48]/46,XN[8] | 85.7 | no abnormalities detected | termination of pregnancy |
| 10 | 17 | 47,XN,+21[20]/46,XN[52] | 27.8 | no abnormalities detected | termination of pregnancy |
| 11 | 20 | 47,XN,+21[16]/46,XN[84] | 16.0 | no abnormalities detected | termination of pregnancy |
| 12 | 18 | 47,XN,+13[6]/46,XN[75] | 7.4 | decreased left heart ratio, narrowed ascending aorta and aortic arch, persistent left superior vena cava, mild tricuspid regurgitation | termination of pregnancy |
| 13 | 19 | 47,XN,+21[15]/46,XN[38] | 28.3 | no abnormalities detected | termination of pregnancy |
| 14 | 18 | 47,XN,+9[5]/46,XN[45] | 10.0 | facial dysmorphism, short limbs, myocardial thickening | termination of pregnancy |
| 15 | 19 | 47,XN,+8,inv(9)(p12q13)[4]/46,XN,inv(9)(p12q13)[46] | 8.0 | NT 4.0mm, hypoplastic left heart, functional single ventricle, ventricular septal defect | live birth |
| 16 | 21 | 47,XN,+i(18)(p10)[22]/46,XN[38] | 36.7 | no abnormalities detected | termination of pregnancy |
| 17 | 20 | 47,XN,+7[8]/46,XN[42] | 16.0 | no abnormalities detected | live birth |
| 18 | 23 | 46,XN,del(20)(p11.2)[18]/46,XN[45] | 28.6 | no abnormalities detected | live birth |
| 19 | 17 | 47,XN,+9[9]/46,XN[88] | 9.3 | left ventricular echogenic focus | lost to follow-up |
| 20^a^ | 13 | 47,XN,+7[15]/46,XN[75] | 16.7 | left choroid plexus cyst | live birth |
|  | 17 | 46,XN | / |  |  |
| 21 | 16 | 47,XN,+9[15]/46,XN[48] | 23.8 | gallbladder not clearly visualized, aberrant right subclavian artery, left ventricular echogenic focus | termination of pregnancy |
| 22 | 19 | 47,XN,+22[33]/46,XN[37] | 47.1 | endocardial cushion defect, focal echogenic bowel (Grade II) | termination of pregnancy |
| 23 | 17 | 46,XN,t(2;13)(q33;p13)[16]/46,XN[74] | 17.8 | no abnormalities detected | live birth |
| 24 | 13 | 47,XN,+9[36]/46,XN[4] | 90.0 | single umbilical artery, mitral and tricuspid regurgitation, dilatation of lateral and third ventricles, retrognathia | termination of pregnancy |
| 25 | 17 | 46,XN,t(6;18)(q25;q21.1)[30]/46,XN[40] | 42.9 | no abnormalities detected | live birth |
| 26 | 18 | 47,XN,+9[10]/46,XN[90] | 10.0 | double superior vena cava | live birth |
| 27 | 13 | 47,XN,+i(7)(p10)[15]/46,XN[35] | 30.0 | left choroid plexus cyst, left ventricular echogenic focus | live birth |
| 28 | 18 | 47,XN,+mar[26]/46,XN[34] | 43.3 | ventricular septal defect (muscular type) | live birth |
| 29 | 22 | 47,XXY[36]/46,XN[4] | 90.0 | / | live birth |
| 30 | 19 | 45,X[42]/47,XXX[12] | 100 | no abnormalities detected | termination of pregnancy |
| 31 | 17 | 45,X[33]/46,XN[30] | 52.4 | / | live birth |
| 32 | 18 | 45,X[4]/46,XN[42] | 8.7 | no abnormalities detected | termination of pregnancy |
| 33 | 32 | 45,X[21]/46,X,del(X)(p22.2)[62] | 100 | fetal growth restriction | termination of pregnancy |
| 34 | 19 | 45,X[38]/46,X,idic(Y)(p11.3)[12] | 100 | no abnormalities detected | termination of pregnancy |
| 35 | 18 | 45,X[55]/46,XN[8] | 87.3 | no abnormalities detected | termination of pregnancy |
| 36 | 15 | 45,X[5]/46,XN[45] | 10.0 | / | live birth |
| 37 | 21 | 45,X[15]/46,XN[32] | 31.9 | no abnormalities detected | termination of pregnancy |
| 38 | 19 | 45,X[10]/46,XN[46] | 17.9 | no abnormalities detected | termination of pregnancy |
| 39 | 18 | 45,X[14]/46,XN[32] | 30.4 | no abnormalities detected | termination of pregnancy |
| 40 | 17 | 45,X[12]/46,XN[36] | 25.0 | no abnormalities detected | lost to follow-up |
| 41 | 18 | 45,X[43]/46,X,del(X)(q25)[7] | 100 | no abnormalities detected | termination of pregnancy |
| 42 | 23 | 47,XXX[28]/46,XN[24] | 53.8 | no abnormalities detected | live birth |
| 43 | 13 | 47,XXX[15]/46,XN[26] | 36.6 | no abnormalities detected | live birth |
| 44 | 22 | 47,XXX[5]/46,XN[41] | 10.9 | no abnormalities detected | lost to follow-up |
| 45 | 26 | 45,X[20]/46,XN[30] | 40.0 | no abnormalities detected | live birth |
| 46 | 18 | 45,X[22]/46,XN[33] | 40.0 | no abnormalities detected | termination of pregnancy |
| 47 | 18 | 45,X[3]/46,XN[32] | 8.6 | no abnormalities detected | live birth |
| 48 | 22 | 47,XXX[12]/45,X[9] | 100 | no abnormalities detected | lost to follow-up |
| 49 | 21 | 45,X[9]/46,XN[46] | 16.4 | no abnormalities detected | live birth |
| 50 | 22 | 45,X[22]/46,XN[28] | 44.0 | aberrant right subclavian artery | termination of pregnancy |
| 51 | 21 | 45,X[3]/46,XN[38] | 7.3 | no abnormalities detected | live birth |
| 52 | 20 | 45,X[5]/46,XN[46] | 9.8 | mild tricuspid regurgitation | live birth |
| 53 | 17 | 47,XYY[35]/46,XN[15] | 70.0 | no abnormalities detected | live birth |
| 54 | 17 | 45,X[10]/46,XN[40] | 20.0 | no abnormalities detected | live birth |
| 55 | 16 | 47,XXX[38]/45,X[22]/46,XN[4] | 93.8 | no abnormalities detected | termination of pregnancy |
| 56 | 20 | 47,XXX[38]/45,X[25] | 100 | no abnormalities detected | live birth |
| 57 | 17 | 45,X[8]/46,XN[72] | 10.0 | no abnormalities detected | live birth |
| 58 | 17 | 47,XYY[52]/46,XN[8] | 86.7 | no abnormalities detected | termination of pregnancy |
| 59^*^ | 12 | 45,X[52]/46,XN[26] | 66.7 | no abnormalities detected | live birth |
|  | 16 | 46,XN | / |  |  |
| 60 | 19 | 45,X[4]/46,XN[56] | 6.7 | no abnormalities detected | lost to follow-up |
| 61 | 18 | 45,X[45]/46,XN[22] | 67.2 | bilateral choroid plexus cysts | lost to follow-up |
| 62 | 22 | 45,X[6]/46,XN[74] | 7.5 | no abnormalities detected | live birth |
| 63 | 16 | 45,X[6]/46,XN[74] | 7.5 | no abnormalities detected | live birth |
| 64 | 17 | 45,X[54]/46,X,+mar[12] | 100 | no abnormalities detected | termination of pregnancy |
| 65 | 13 | 45,X[15]/46,XN[17] | 46.9 | no abnormalities detected | termination of pregnancy |
| 66 | 16 | 46,X,i(X)(q10)[65]/45,X[5] | 100 | no abnormalities detected | termination of pregnancy |
| 67 | 16 | 45,X[5]/46,XN[65] | 7.1 | no abnormalities detected | termination of pregnancy |
| 68 | 17 | 45,X[30]/46,XN[15] | 66.7 | bilateral choroid plexus cysts, Single umbilical artery | termination of pregnancy |
| 69 | 20 | 45,X[11]/46,XN[66] | 14.3 | no abnormalities detected | live birth |
| 70 | 18 | 45,X[16]/46,XN[84] | 16.0 | no abnormalities detected | termination of pregnancy |
| 71 | 18 | 47,XXY[53]/46,XN[17] | 75.7 | no abnormalities detected | live birth |
| 72 | 18 | 45,X[6]/46,XN[94] | 6.0 | no abnormalities detected | live birth |
| 73 | 17 | 45,X[42]/46,XN[8] | 84.0 | ductus venosus not clearly visualized | termination of pregnancy |
| 74 | 16 | 47,XXX,inv(9)(p12q13)[5]/46,XN,inv(9)(p12q13)[35] | 12.5 | no abnormalities detected | live birth |
| 75 | 13 | 45,X[35]/47,XXX[15] | 100 | NT 4.3mm | termination of pregnancy |
| 76 | 12 | 47,XYY[65]/46,XN[5] | 92.9 | NT 4.7mm | termination of pregnancy |
| 77 | 18 | 47,XXY[18]/46,XN[32] | 36.0 | no abnormalities detected | live birth |
| 78 | 21 | 45,X[38]/46,XN[2] | 95.0 | no abnormalities detected | termination of pregnancy |
| 79 | 20 | 47,XXY[56]/46,XN[8] | 87.5 | no abnormalities detected | live birth |
